# Scleral remodeling during myopia development in mice eyes: a potential role of thrombospondin-1

**DOI:** 10.1186/s10020-024-00795-x

**Published:** 2024-02-14

**Authors:** Junhan Chen, Shin-ichi Ikeda, Yajing Yang, Yan Zhang, Ziyan Ma, Yifan Liang, Kazuno Negishi, Kazuo Tsubota, Toshihide Kurihara

**Affiliations:** 1 Laboratory of Photobiology, Keio University School of Medicine, 35 Shinanomachi, Shinjuku-ku, Tokyo, 160-8582 Japan; 2 Department of Ophthalmology, Keio University School of Medicine, 35 Shinanomachi, Shinjuku-ku, Tokyo, 160-8582 Japan; 3 Tsubota Laboratory, Inc, 34 Shinanomachi, Shinjuku-ku, Tokyo, 160-0016 Japan

**Keywords:** Myopia, Sclera, ECM, THBS1

## Abstract

**Background:**

Scleral extracellular matrix (ECM) remodeling plays a crucial role in the development of myopia, particularly in ocular axial elongation. Thrombospondin-1 (THBS1), also known as TSP-1, is a significant cellular protein involved in matrix remodeling in various tissues. However, the specific role of THBS1 in myopia development remains unclear.

**Method:**

We employed the HumanNet database to predict genes related to myopic sclera remodeling, followed by screening and visualization of the predicted genes using bioinformatics tools. To investigate the potential target gene *Thbs1*, we utilized lens-induced myopia models in male C57BL/6J mice and performed Western blot analysis to detect the expression level of scleral THBS1 during myopia development. Additionally, we evaluated the effects of scleral THBS1 knockdown on myopia development through AAV sub-Tenon’s injection. The refractive status and axial length were measured using a refractometer and SD-OCT system.

**Results:**

During lens-induced myopia, THBS1 protein expression in the sclera was downregulated, particularly in the early stages of myopia induction. Moreover, the mice in the THBS1 knockdown group exhibited alterations in myopia development in both refraction and axial length changed compared to the control group. Western blotting analysis confirmed the effectiveness of AAV-mediated knockdown, demonstrating a decrease in COLA1 expression and an increase in MMP9 levels in the sclera.

**Conclusion:**

Our findings indicate that sclera THBS1 levels decreased during myopia development and subsequent THBS1 knockdown showed a decrease in scleral COLA1 expression. Taken together, these results suggest that THBS1 plays a role in maintaining the homeostasis of scleral extracellular matrix, and the reduction of THBS1 may promote the remodeling process and then affect ocular axial elongation during myopia progression.

**Supplementary Information:**

The online version contains supplementary material available at 10.1186/s10020-024-00795-x.

## Introduction

Myopia, commonly known as nearsightedness, is a refractive error that impairs clear vision of distant objects (Baird et al. 2020). Over the past half-century, the global prevalence of myopia has significantly increased (Holden et al. 2016). Furthermore, it is expected that nearly 10 million individuals will have high myopia, which is defined as a refractive error exceeding − 6.00 diopters (Sankaridurg et al. 2021). High myopia is associated with a higher susceptibility to retinal abnormalities and visual impairment, including conditions such as retinal detachment, cataracts, glaucoma, and myopic maculopathy. The progression of myopia is believed to involve extracellular matrix (ECM) remodeling of the sclera, leading to irreversible deformation and axial elongation of the eye (Harper and Summers 2015). However, the precise mechanisms underlying the onset and development of ECM remodeling and axial elongation in myopia remain unclear.

Numerous studies have explored the relationship between scleral ECM genes and myopia. Collagen genes, tissue inhibitors of metalloproteinases (*TIMPs*), and matrix metalloproteinases (*MMPs*), which regulate the degradation of collagen and other ECM components, have garnered significant attention in myopia research (Yang et al. 2009; Jia et al. 2017). Nevertheless, despite the identification of approximately 1027 ECM-related genes in humans, the exact function of scleral ECM in myopia development requires further investigation (Naba et al. 2012). Bioinformatics tools enable researchers to discover potential functional genes by combining large amounts of existing data and have been widely used in ophthalmology research fields including ECM and myopia (Mei et al. 2017; Hu et al. 2021; Mo et al. 2021). The development of new bioinformatics tools and databases brings more possibilities for finding genes related to myopic sclera remodeling.

*Thbs1*, also known as *Tsp1*, is a critical extracellular matrix protein that mediates ECM remodeling and enhances ECM homeostasis (Rosini et al. 2018). Previous studies have demonstrated a decreased expression of THBS1 during the development of myopia in a lens-induced myopia model, suggesting its involvement in scleral ECM remodeling (Gao et al. 2011; Guo et al. 2013, 2014). But the role of THBS1 in scleral remodeling remains to be investigated. Here, we reported that that lens-induced mouse myopia (LIM) induced the downregulation of scleral THBS1, thereby confirming its potential role in mediating scleral ECM remodeling and ocular axial elongation in mice.

## Materials and methods

### Prediction of myopia-associated ECM genes

To predict myopia-associated ECM genes, previously reported genes were identified through a literature review and the Consortium of Refractive Error and Myopia (CREAM) report, serving as guide genes (Haarman et al. 2021). These guide genes were used to search for novel candidate genes by leveraging the direct linkages of HumanNet V3, an integrated network of human genes for disease studies based on supervised machine learning techniques (Kim et al. 2022). For the inference algorithm model, we selected HumanNet-XC, which incorporates advanced models such as co-functional links, co-citation, co-expression, pathway database, domain profile association, genetic interaction, gene neighborhood, phylogenetic profile association, and protein-protein interaction (PPI) network predictions. HumanNet-XC has connected 18,462 genes with 1,125,494 connections, enabling researchers to gain a comprehensive understanding of complex biological processes and identify potential disease-related genes. To assess the discriminative power of gene prediction, we performed Area under the Receiver Operating Characteristic Curve (AUROC) analysis which was based on HumanNet database and exported the top 5% ranked potential genes for further analysis. AUROC represents the prediction power of candidate genes in top ranks.

### Functional enrichment analysis and validation of hub genes

To validate the association of the gene list with myopic scleral matrix remodeling again, we performed Reactome pathway and functional enrichment analyses using g: Profiler and visualization tools based on the R platform’s clusterProfiler package (Raudvere et al. 2019; Li et al. 2022). The gene list obtained from the inference algorithm was imported into the Search Tool for the Retrieval of Interacting Genes/Proteins (STRING), a database that combines known and predicted protein-protein interactions (Szklarczyk et al. 2021). STRING incorporates direct and indirect associations derived from computational prediction, knowledge transfer between organisms, and aggregation from other primary databases. We exported the PPI network as a TSV document and reconstructed it using Cytoscape software (Kohl et al. 2011). To rank the nodes in the predicted myopia ECM network, we utilized the cytoHubba plugin in Cytoscape, which combines multiple algorithms to determine the importance of nodes in the overall network (Chin et al. 2014). Based on previous literature reports, we employed the Maximal Clique Centrality (MCC) algorithm, which has demonstrated better accuracy performance among the 11 algorithms available in cytoHubba, to sort the potential hub genes (Chin et al. 2014). In this network, nodes with higher MCC algorithm scores have higher centrality. The centrality of each node was represented by shades of color, with darker colors indicating higher values.

### Animal administration

Male C57BL6J mice were housed in a controlled environment consisting of standard transparent cages maintained at a temperature of 24 ± 2 °C and a humidity range of 40–60%. The mice were kept in a clean room operating on a 12-hour light-dark cycle. During the entire experimental period, the mice were provided ad libitum access to a standard rodent diet and water.

Ethical approval for all animal experiments conducted in this study was obtained from the Animal Experimental Committee of Keio University (A2022-242). The study adhered to the Institutional Guidelines on Animal Experimentation at Keio University, the ARVO Statement for the Use of Animals in Ophthalmic and Vision Research, and the Animal Research: Reporting of In Vivo Experiments (ARRIVE) guidelines for the use of animals in research.

### Establishment of LIM and measurement of ocular biometric characteristics in mice

**Fig. 1:**
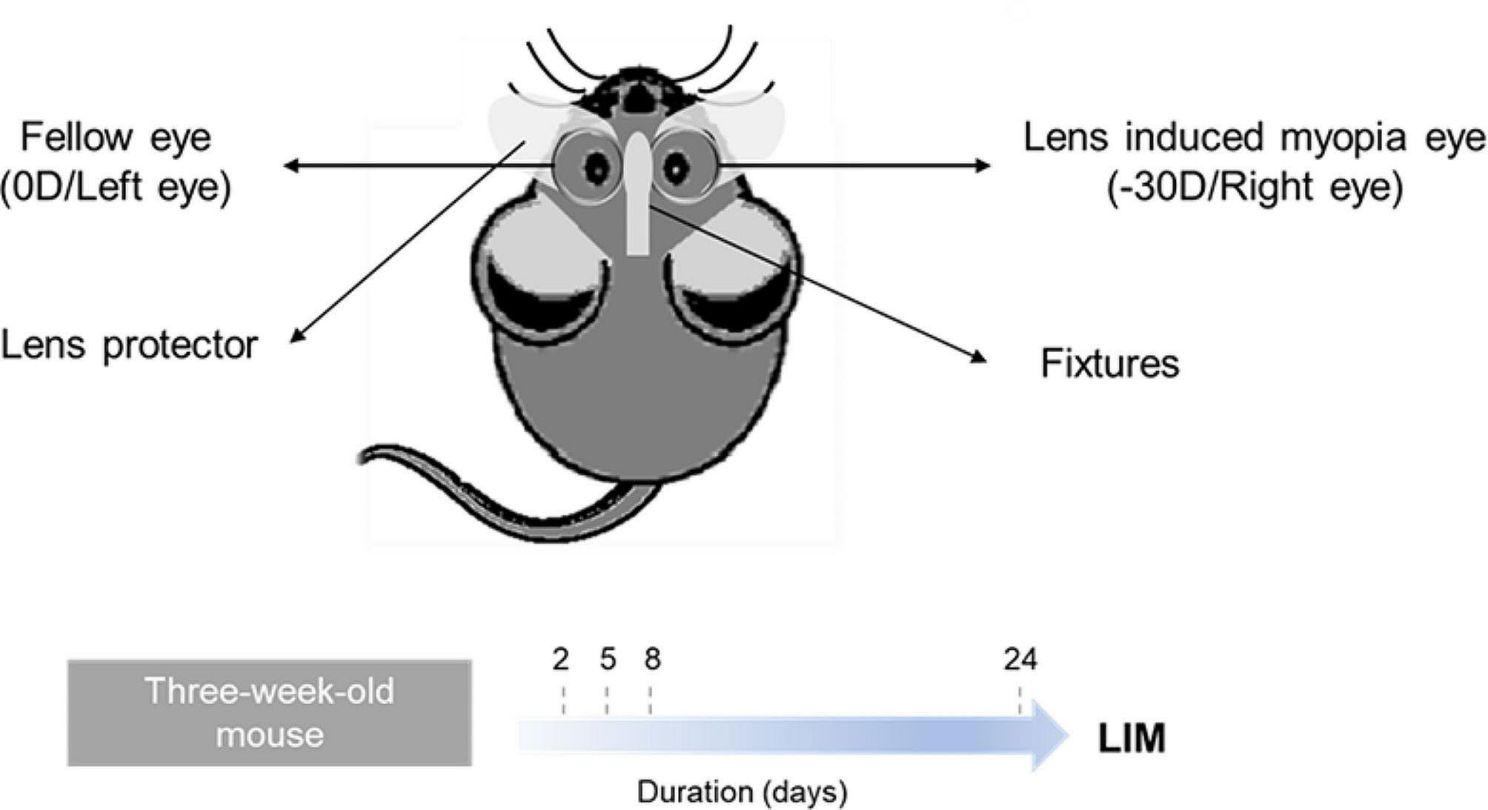
Construction of lens-induced myopia (LIM) mice models. Monocular LIM was induced by -30D lens attached in the right eyes while 0D lens with left eyes as the internal control. The time course of LIM started from three-week-old mice and lasted 2 days, 5 days, 8 days and 24 days

According to previous reports by our laboratory, monocular LIM was induced in mice (Jiang et al. 2018). The left side of the glasses used in this study was affixed with a 0D lens as an internal control, and the right side of the glasses was affixed with a -30D lens (Fig. 1).

The glasses were removed and washed at least twice a week. Ocular biometric characteristics, including the refractive state measured using an infrared photorefractor (Steinbeis Transfer Center) and axial length (AL) and choroid thickness analyzed using an SD-OCT system (Envisu R4310, Leica) designed for mice, were measured according to previous reports.

In this study, a time-course LIM operation was performed on male wildtype C57BL/6J mice at 3 weeks of age for durations of 2 days, 5 days, 8 days, and 24 days. 3-week-old male C57BL/6 mice (*n* = 20) were randomly divided into 4 groups of different durations of LIM.

### AAV-based CRISPR/Cas9 system for THBS1 disruption in mouse sclera

SaCas9 or guide RNA (Supplementary Table T2) against THBS1 expression cassettes were constructed and packaged into AAV-DJ vectors (titer > 1.0E + 13 GC/ml) by Vector Builder as described in previous reports (Ikeda et al. 2022; Zhao et al. 2018). The mice were randomly assigned to three groups: a sham-operation group (anaesthetized and had a ‘dry’ needle inserted with no injection), a group injected with scramble guide RNA, and a group injected with gThbs1. A 1:1 mixture of AAV-packaged SaCas9 and guide RNA was injected into mice following anesthesia and the application of Scopisol to prevent reflux. Subtenon’s injections were administered at two locations around each eye, spaced 180 degrees apart (while avoiding blood vessels) as previous reported (Supplementary Figure S1) (Ikeda et al. 2022; Zhao et al. 2018). A 33 G needle was used for the injections, and the eyes were covered with 0.2% polyacrylic acid for the 3 days following the injection.

To evaluate the efficacy of subtenon’s AAV injection for gene transfer into scleral tissue, the expression of green fluorescent protein (GFP) in sclera flat mounts was checked 28 days after AAV-DJ-GFP or AAV-DJ-Vector injection using a Keyence BZ-800 fluorescence microscope.

### Western blotting

After administering anesthesia, mice were euthanized, and two sclera samples were extracted from each mouse. Subsequently, the two sclera samples obtained from a single mouse were pooled to create a unified sample. Each lane of western blot corresponded to a different independent sample. Sclera samples were homogenized in RIPA buffer supplemented with Halt protease inhibitor cocktail (ThermoFisher Scientific, USA). Protein concentration was determined using the BCA protein assay, and samples were adjusted with Laemmli sample buffer (Nacalai Tesque, Japan). The protein extracts were separated by SDS-PAGE, transferred to PVDF membranes (Merck Millipore, MA, USA), and blocked with Blocking One (Nacalai Tesque, Japan). Membranes were then incubated overnight at 4 °C with specific antibodies, followed by incubation with corresponding secondary antibodies. Visualization was performed using SuperSignal West Femto Maximum Substrate (Thermo Fisher Scientific, USA). SDS-PAGE was conducted on 10% acrylamide gels with protein size markers (MagicMark XP Western Protein Standard, Thermo Fisher Scientific, USA). The primary and secondary antibodies used for western blot were listed as follows: THBS1 (1:1000 dilution, Invitrogen, #39-9300), COL1A1 (1:1000 dilution, Cell Signaling Technologies, #84,336), LRP1 (1:1000 dilution, Cell Signaling Technologies, #64,099), TGFB1 (1:1000 dilution, Abcam, ab179695), MMP9 (1:1000 dilution, Abcam, ab228402), α-SMA (1:100 dilution, Invitrogen, #14-9760-82), and β-ACTIN (1:5000 dilution, Cell Signaling Technologies, #3700).

### Statistical analysis

To determine the statistical significance of comparisons, independent sample t-tests (Fig. 4) and analysis of variance (ANOVA) with Tukey post hoc test (Figs. 5 and 6) were performed individually using GraphPad Prism 9. Figures 4, 5 and 6 are average of experiments and Fig. 7 is representative or of experiments. Choroidal thickness and histogram analysis of Western blot were conducted using Image J (version 1.53t; NIH). A significance level of *P*-value < 0.05 was considered statistically significant.

## Results

### ECM genes predicted by inference algorithm model

We obtained 44 previous reported myopia-associated ECM genes from literature review and CREAM report as guide genes (Table 1). The HumanNet database creates null models by generating 10,000 random gene sets of equivalent size to assess the statistical significance of the observed AUROC score. The ROC analysis resulted in an AUROC = 0.9424, indicating that the prediction of HumanNet V3 has excellent resolution ability (Supplementary Figure S2). The initial list was arranged according to the order presented in Supplementary Table 1, which was scored by the dtabase. A total of 3002 genes were predicted and ranked based on their scores, and the top 5% genes were selected for further analysis with similar strategies employed in previous studies (Taneera et al. 2012; Stokes and Visweswaran 2012).

**Table 1:** Previous reported myopia associated ECM genes

Guide genes
ADAMTS2, ADAMTS10, ADAMTS17, ADAMTS18, ADAMTSL1, ADAMTSL4, BMP2, BMP3, BMP4, BMP6, CCL4, COL2A1, COL4A1, COL4A3, COL4A5, COL5A1, COL6A1, COL8A1, COL9A1, COL9A2, COL10A1, COL11A1, EFEMP1, FBN1, FBN2, GPC5, GPC6, IFNB1, IL23A, KAZALD1, LAMA2, LTBP2, NRG1, PLOD1, PLOD3, PZP, RSPO1, SEMA3D, SEMA4F, TNFSF12, TNFSF13, VCAN, WNT7B.
The list contained 44 previous reported myopia associated ECM genes, obtained from literature review and CREAM report.

### Functional enrichment analysis of ECM genes

**Fig. 2:**
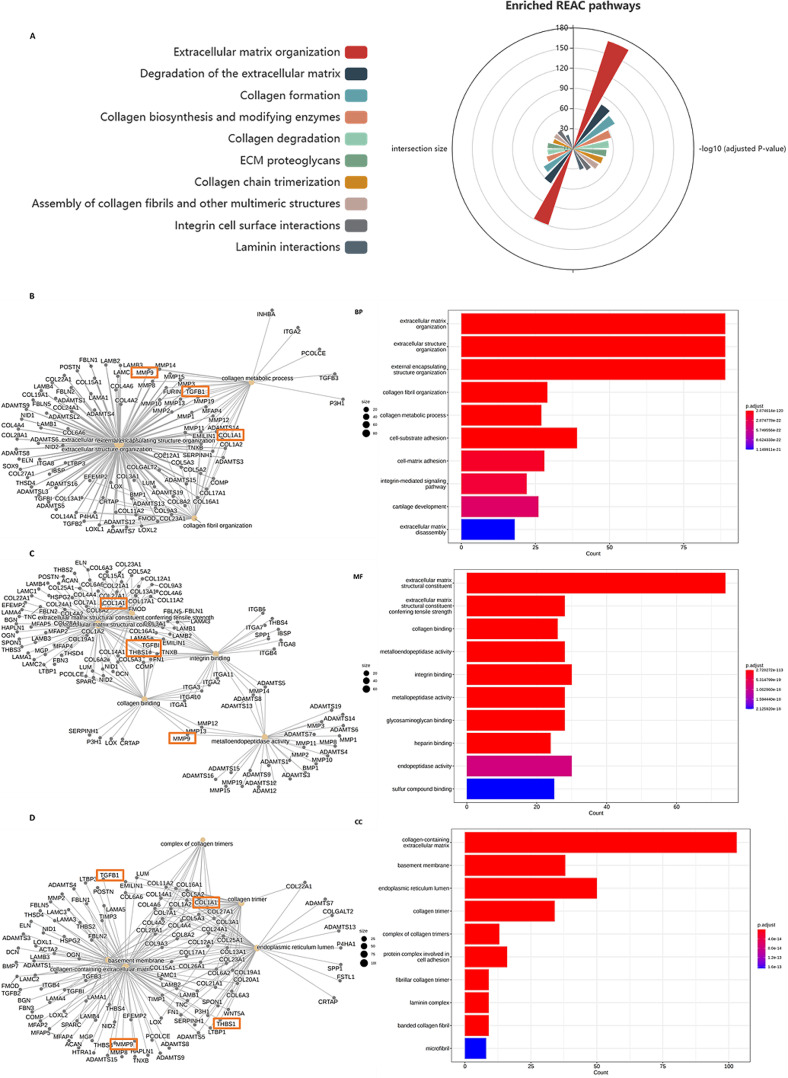
Results of Reactome pathway and Gene Ontology enrichment analysis. **A**: Polar plot of top ten enriched Reactome pathways of potential myopia associated ECM genes with maximum adjusted P-value of 1.73E-33. **B**: Top ten gene ontology biological process (BP) annotations with maximum adjusted P-value of 1.15E-21. **C**: Top ten gene ontology molecular function (MF) annotations with maximum adjusted P-value of 2.13E-18. **D**: Top ten gene ontology cellular component (CC) annotations with maximum adjusted P-value of 1.60E-13

To analyze the functional characteristics of the ECM genes, we performed Reactome pathway analysis and Gene Ontology annotation using the g: Profile database and visualization tools based on the R platform. Nineteen Reactome pathways were significantly enriched with *P* = 1.0E-20 as the cutoff. Among them, the top three Reactome pathways were “Extracellular matrix organization, P = 2.203E-169”, “Degradation of the extracellular matrix, P = 3.647E-79” and “Collagen formation, P = 2.866E-73”. The top three terms under Gene Ontology Biological Process were “extracellular matrix organization, P = 2.875E-120”, “extracellular structure organization, P = 2.875E-22” and “external encapsulating structure organization, P = 5.749E-22”. At the same time, gene ontology molecular function (MF) and cellular component (CC) analyses were also performed (see details of top 10 terms of each analysis in Fig. 2). These results demonstrate a strong correlation between the predicted gene list and the composition, homeostasis, and remodeling of the ECM matrix, further confirming the discriminative power of the prediction tool by HumanNet V3.

### Construction of PPI network and identification of hub genes

**Fig. 3:**
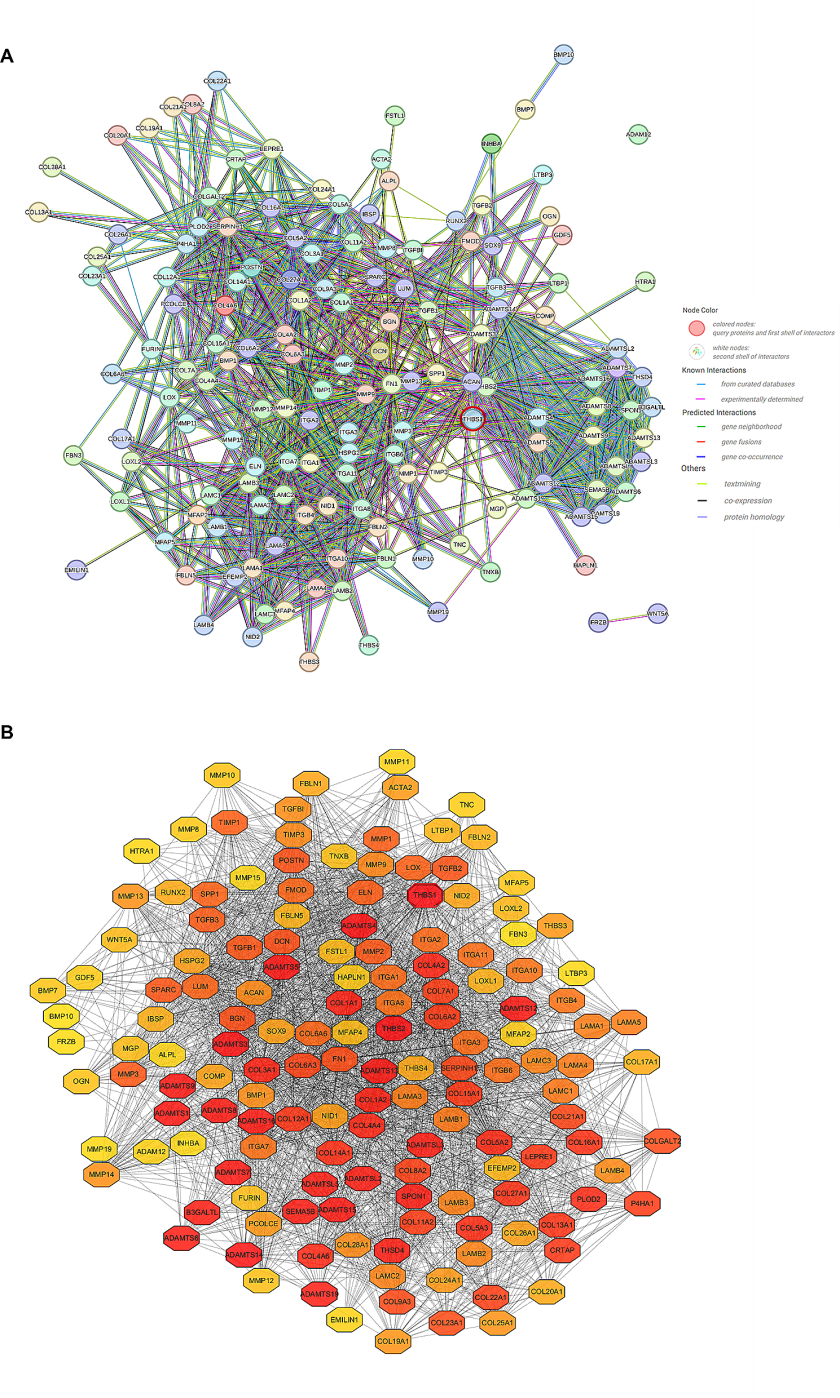
Visualization of the overall network. **A**: Protein-protein interaction network of high confidence score (0.700) by STRING. The network nodes represent the proteins corresponding to the genes, and the edges represent the possible protein interactions. The different connection colours indicate specific types of interactions. **B**: The visualized result of the gene-interaction network by Cytoscape. The network nodes represent the proteins corresponding to the genes, and the edges represent the protein interactions. The node’s shading, ranging from light to dark, corresponds to their MCC score, nodes with darker shades indicate stronger connections within the network

A PPI network was constructed in STRING with minimum required interaction score = 0.7 of high confidence (Fig. 3a). The result of STRING network then visualized by Cytoscape as TSV document, which included 150 nodes and 5788 edges. CytoHubba was used to analyze the centrality of individual nodes in the overall network based on the selected MCC algorithm (Fig. 3b). In this analysis, THBS1 had the highest score (1.13E + 21) among the gene nodes, suggesting that THBS1 may play a potential role in scleral ECM remodeling.

### Establishment of time-course LIM models and scleral THBS1 expression level during myopia development

**Fig. 4:**
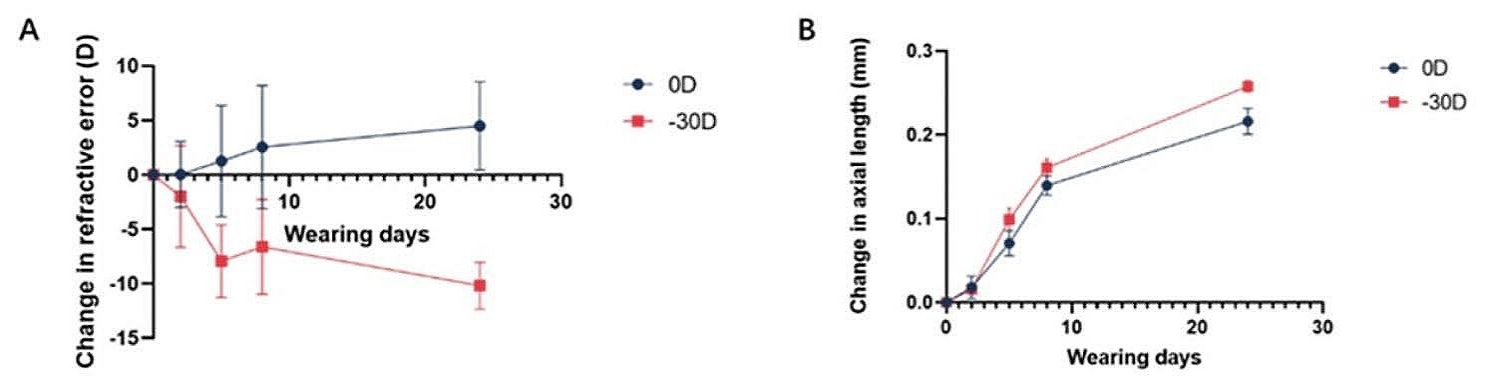
Ocular biometric characteristics of time-course LIM mice. **A**: Change in refractive error during 2/5/8/24-days LIM in C57BL6J mice. D, diopter. 0D: left eyes affixed with a 0D lens as an internal control; -30D: right eyes affixed with a -30D lens as monocular LIM. Bars represent mean ± standard deviations. **B**: Change in axial length during 2/5/8/24-days LIM in C57BL6J mice. 0D: left eyes affixed with a 0D lens as an internal control; -30D: right eyes affixed with a -30D lens as monocular LIM. Bars represent mean ± standard deviations. Each group *n* = 5 (the total sample size of the four groups *n* = 20), figure is an average of experiments

From the fifth day onwards, significant differences in both change in refractive error (Fig. 4a) and change in axial length (Fig. 4b) were observed between the LIM/-30D and control/0D eyes.

**Fig. 5:**
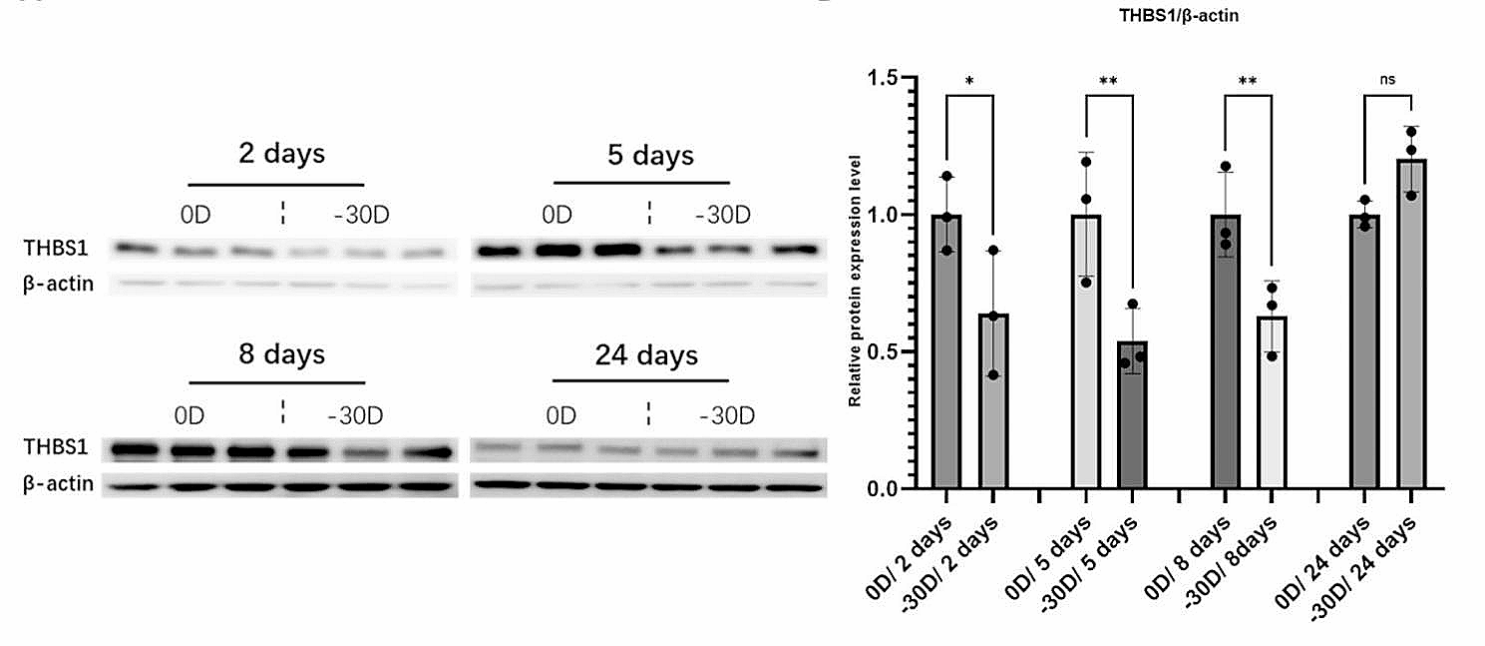
THBS1 expression level during the development of myopia induced by LIM. **A**: After LIM induction for 2/5/8/24 days, western blot was performed to show the change of protein expression level of THBS1. The − 30D eyes and the 0D eyes with the same duration of LIM were used for comparison. **B**: Densitometric quantification of the blot using ImageJ. Each group *n* = 3, figure is an average of experiments. Student’s two-tailed t-test, *p < 0.05, **p < 0.01. Bars represent mean ± standard deviations

At the end of each LIM group, samples of the sclera were collected and used for western blotting analysis. The results of western blotting showed that compared to the control eyes (wearing 0D lenses), the LIM eyes (-30D) exhibited a decrease in the expression levels of the THBS1 in sclera samples collected for 2 days/5 days/8 days (Fig. 5).

### Scleral THBS1 knockdown induced axial elongation

**Fig. 6:**
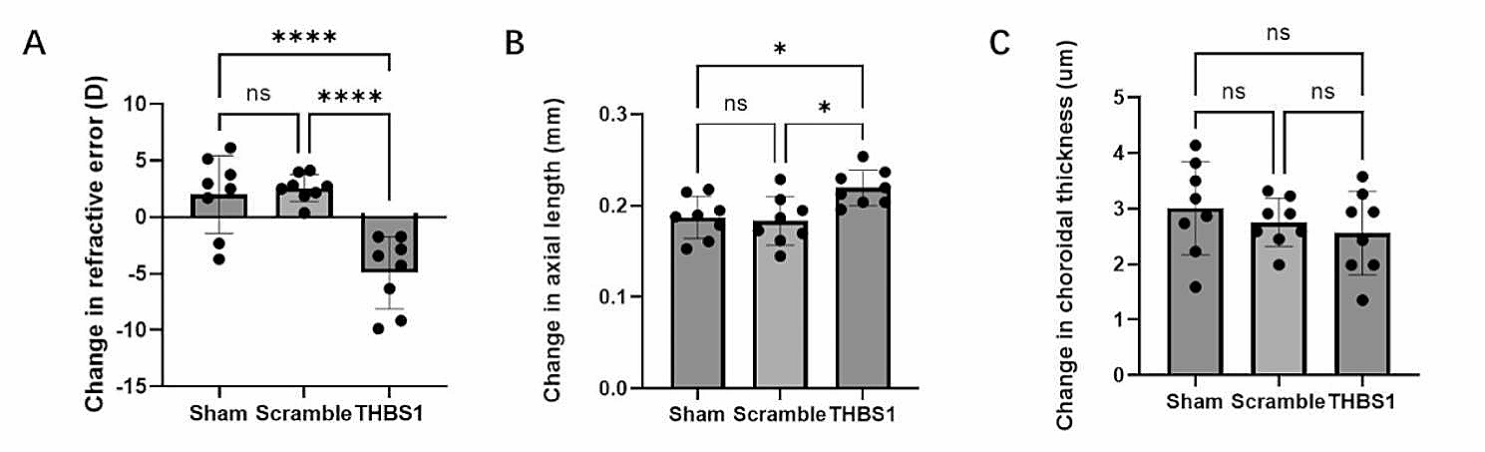
Scleral THBS1 knockdown induced axial elongation and myopic refraction shift. **A**: Myopic refractive change was observed in mice of the THBS1 knockdown group at 28 days after AAV injection but not in Sham and Scramble groups. D, diopter. Each group *n* = 8, figure is an average of experiments, one-way ANOVA with Tukey post hoc test, ****p < 0.0001, NS: no significance. Bars represent mean ± standard deviations. **B**: Mice of THBS1 knockdown group showed more axial elongation than control group at 28 days after AAV injection. Each group *n* = 8, figure is an average of experiments, one-way ANOVA with Tukey post hoc test, *p < 0.05, NS: no significance. Bars represent mean ± standard deviations. **C**: No significant difference of choroidal thickness was found between the THBS1 knockdown, Sham or Scramble groups at 28 days after AAV injection. Each group *n* = 8, figure is an average of experiments, one-way ANOVA with Tukey post hoc test, NS: no significance. Bars represent mean ± standard deviations

To assess the effectiveness of subtenon’s AAV injection for gene transfer into scleral tissue, we examined GFP expression in sclera flat mounts 28 days post AAV-DJ-GFP or AAV-DJ-Vector injection. GFP distribution was evident in eyes injected with AAV-DJ-GFP, whereas no obvious GFP expression was observed in eyes injected with AAV-DJ-Vector (Supplementary Figure S3). To investigate the possible role of THBS1 in sclera ECM, we performed scleral-specific THBS1 knockdown using CRISPR/Cas9 system. Expression vector of S*taphylococcus aureus* Cas9 (SaCas9) and guide RNA against *Thbs1* were delivered two separate adeno-associated virus (AAV, serotype: DJ) through co-injection into sub-Tenon’s capsule in both eyes, along with scrambled, non-targeting guide RNA and sham group control (*n* = 8). After 24 days of AAV injection, g*Thbs1* injected showed more axial elongation and refractive error compared to Scramble and Sham groups, but not evidently change in choroidal thickness assessed by previous reported method (Jeong et al. 2023) (Fig. 6).

**Fig. 7:**
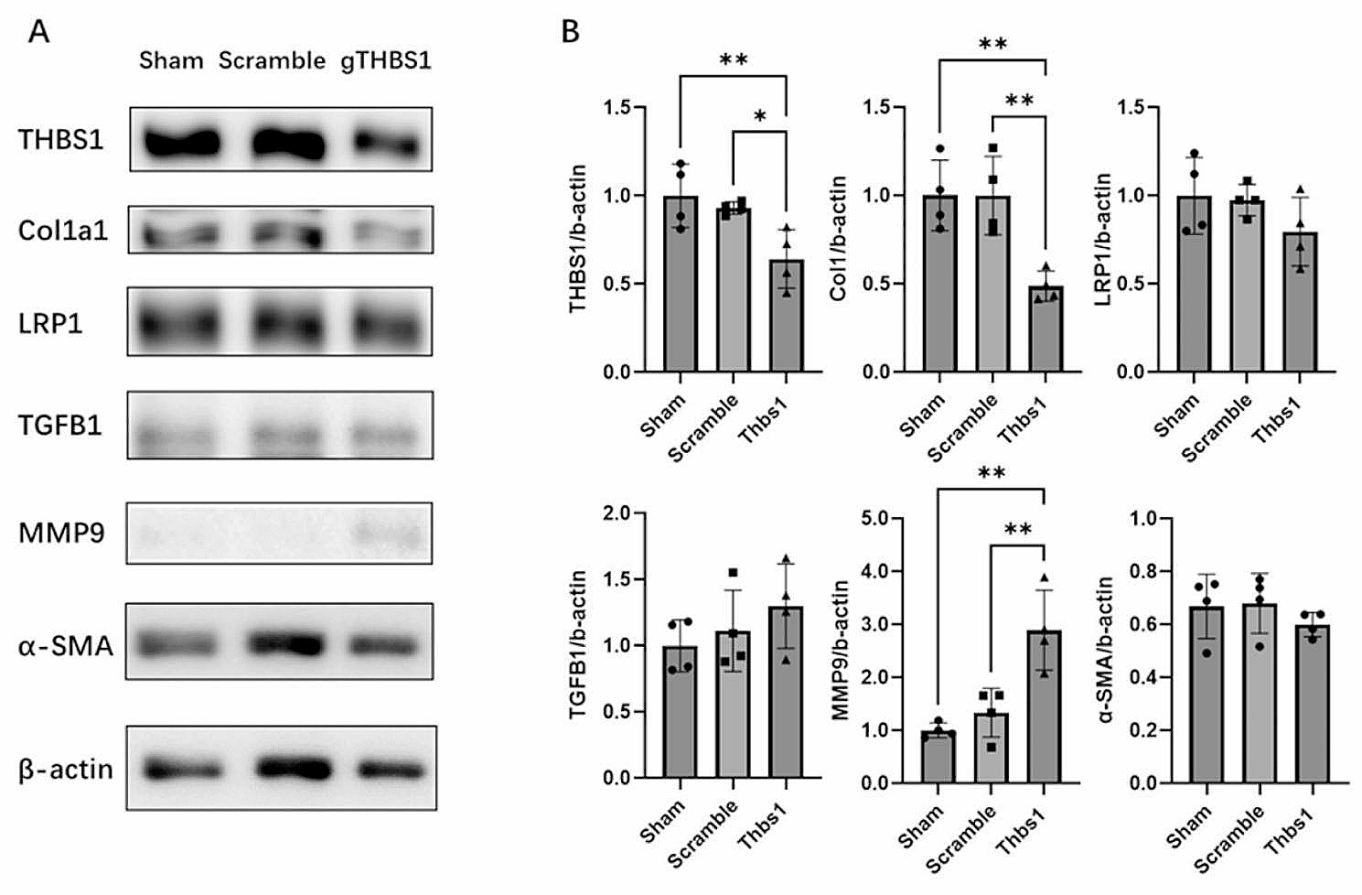
Western blotting results of sclera samples for THBS1 and potential interacted factors. **A**: Western blot showed the change of protein expression level of THBS1 and its potential downstream factors at 28 days after AAV injection. **B**: Densitometric quantification of the blot in Fig. 6a using ImageJ. Each group *n* = 4, figure is representative of experiments (a total of four replicates of independent samples were used for the analysis), one-way ANOVA with Tukey post hoc test, *p < 0.05, **p < 0.01. Bars represent mean ± standard deviations

To confirm the knockout of THBS1 in the sclera and explore the mechanism of THBS1-mediated sclera remodeling, sclera samples were collected and analyzed by western blotting. Previous studies have suggested that THBS1 can regulate collagen homeostasis through collagen decomposition and generation (Rosini et al. 2018). We checked several previous reported *Thbs1* related genes, and our western blotting results showed that compared to the Scramble and Sham groups, the g*Thbs1* group exhibited evidently decreased expressions of THBS1 and COL1A1 in the sclera, while the expression of MMP9 was significantly increased. However, we did not observe significant differences in the expression levels of LRP1, TGFB1 and α-SMA among the three groups (Fig. 7).

## Discussion

Scleral ECM has been confirmed to be closely associated with the occurrence and progression of myopia (Yu and Zhou 2022). The normal thickness and elasticity of the sclera are crucial for maintaining the mechanical stability of the eye and safeguarding internal structures against external damage (McBrien et al. 2009). Remodeling of the scleral ECM can impact the biomechanical properties of the sclera, and any alterations in its mechanical properties may lead to refractive errors (McBrien et al. 2009). Recent studies have indicated that hypoxia might be one of the key mechanisms inducing abnormal ECM remodeling in the regulation of scleral ECM during myopia (Wu et al. 2018). Additionally, our previous findings demonstrated that endoplasmic reticulum stress in the sclera might play a significant role in the expression of ECM proteins (Ikeda et al. 2022). However, due to the intricate composition of the ECM, elucidating the role of specific ECM genes in the myopia process is challenging, and further exploration of the associated mechanisms is warranted.

In this study, we initially generated a preliminary list of candidate genes using a machine learning algorithm-based prediction model (Kim et al. 2022). While the results of the ROC analysis confirmed the model’s high discriminative power, considering the broad range of candidate gene lists obtained from the inference model (scores ranging from 82.3 to 1.46), we further refined the gene list and constructed a PPI network to revalidate the correlation between the resulting gene list and ECM remodeling, as well as identify the central node. Enrichment analysis revealed a strong correlation between candidate gene networks and ECM remodeling. For the hub gene screening method, we chose the MCC algorithm, as it captures more essential proteins in the top-ranked list of high- and low-level proteins, demonstrating better performance based on previous reports (Chin et al. 2014). Among the genes, *Thbs1* exhibited the highest MCC score in the overall gene network and was selected for further evaluation. Notably, *Thbs2*, a related gene belonging to the thrombospondin family, ranked second, sharing considerable similarities with *Thbs1* while possessing distinct characteristics (Bornstein et al. 2004). Although we did not explore its role in regulating scleral ECM in this study, it could be an intriguing direction for future research.

Our in vivo studies demonstrated downregulation of THBS1 expression levels in the sclera of lens-induced myopia during myopia progression, consistent with previous reports in other animal models (Gao et al. 2011; Guo et al. 2013). In the early stage of myopia induction, THBS1 expression decreased, and this trend continued before day 24 (relatively stable stage of myopia induction). To mitigate potential influences, we used AAV-mediated sclera THBS1 knockout instead of THBS1 global knockout mice, as THBS1 global knockout mice develop severe dry eye, akin to autoimmune disease Sjogren’s syndrome, which may not be suitable for studying myopia models (Turpie et al. 2009). In the knockdown experiments, reduction of scleral THBS1 through AAV-DJ gene transfer induced axial elongation in mice eyes, consistent with a previous study, suggesting a potential role of THBS1 in scleral ECM remodeling (Hu et al. 2021).

Previous studies have reported the regulatory role of THBS1 on collagen homeostasis via the TGF-β pathway and interaction with different matrix metalloproteinases (MMPs) (Rosini et al. 2018; Patwardhan et al. 2021; Yamashiro et al. 2020). Our results indicated a significant decrease in the expression level of COL1A1, consistent with findings observed in multiple myopia models (Gentle et al. 2003). THBS1 is a known activator of TGF-β1 in vivo (Lopez-Dee et al. 2011) and TGF-β1 pathway has been determined as one of the most important pathways during myopia development (McBrien 2013; Jobling et al. 2008; Meng et al. 2015; Zha et al. 2009). Previous studies reported that TGF-β1 can induce THBS1 expression and act on ECM remodeling (Pal et al. 2016; Hsieh 2019). Though the total amount of TGF-β1 showed no significant change, it’s reasonable that knockdown of THBS1 may affect the activation of latent TGF-β1 and thus alter related pathways (Farberov and Meidan 2016; Ahamed et al. 2009). There is another possibility that this discrepancy is due to the detection time point, similar to a previous study where THBS1 knockdown failed to significantly alter TGFB protein expression (Farberov and Meidan 2014). Further experiments will be needed to better understand the THBS1-TGF-β1 signaling in scleral fibroblasts. At the same time, it is well-established that THBS1 can regulate MMP9 expression and activation through multiple pathways (Sfar et al. 2007). According to our results, THBS1 knockdown led to a significant increase in MMP9 expression, consistent with some previous studies (Ren et al. 2019; Kim et al. 2013; Nucera et al. 2010). Interestingly, some other reports have shown that although THBS1 can inhibit MMP9 activity, the expression of THBS1 is positively correlated with MMP9, possibly due to different cell and tissue types (Yin et al. 2019; Albo et al. 2002). In sum, while our study elucidates THBS1’s potential contributions to collagen homeostasis and its involvement in modulating MMP9 expression, we acknowledge that several facets warrant further investigation and clarification. The intricate interplay between THBS1, TGF-β1, and MMP9 within the context of scleral remodeling demands more extensive exploration to unravel the mechanisms that underlie myopia development. Further studies, such as in vitro experiments using scleral fibroblasts, are needed to fully reveal the complex mechanism by which THBS1 acts in the sclera.

Previous reports indicated that rhTHBS1 treatment of primary fibroblasts stimulated collagen1 production in a dose-dependent manner (Hsieh 2019). This observation raises the prospect of developing myopia treatments with THBS1 as a target. Furthermore, it is noteworthy to mention a previous study that observed an upregulation of THBS1 expression in the sclera of guinea pigs upon exposure to short-wavelength light, specifically at a peak value of 440 nm with a half bandwidth of 20 nm (Wen et al. 2021). Our previous research indicated that violet light (360 to 400 nm) can suppress myopia development, making it intriguing to explore the potential connection between THBS1 and these findings (Jiang et al. 2021).

## Conclusion

In summary, our study suggests a potential role for THBS1 in mediating scleral ECM remodeling during myopia development. Notably, the decreased expression of scleral THBS1 is observed at the onset of lens-induced myopia (LIM) induction. This downregulation may initiate a cascade of events leading to scleral extracellular matrix remodeling conducive to myopia development. The remodeling process may include the upregulation of MMP9 and the facilitation of collagen I degradation.

### Electronic supplementary material

Below is the link to the electronic supplementary material.

Supplementary Material 1

## Data Availability

The data that support the findings of this study are available in the body of the text and supplemental materials.

## Abbreviations

ECM: Extracellular matrix
THBS1: Thrombospondin-1
AAV: Adeno-associated virus
Col1A1: Collagen 1A1
TIMPs: Tissue inhibitors of metalloproteinases
MMPs: Matrix metalloproteinases
PPI: Protein-protein interaction
STRING: Search Tool for the Retrieval of Interacting Genes/Proteins
MCC: Maximal Clique Centrality
ARRIVE: ARVO Statement for the Use of Animals in Ophthalmic and Vision Research, and the Animal Research
ANOVA: Analysis of variance
BP: Biological process
MF: Molecular function
CC: Cellular component
GFP: Green fluorescent protein
AL: Axial length
CREAM: Consortium of Refractive Error and Myopia
AUROC: Area under the Receiver Operating Characteristic Curve
