# Supplementary material for: Scleral remodeling during myopia development in mice eyes: a potential role of thrombospondin-1
Source: Mol Med. 2024 Feb 14;30:25. doi: 10.1186/s10020-024-00795-x (PMC10865574; doi:10.1186/s10020-024-00795-x)
Supplement: Supplementary file 1 — Supplementary Material 1 [file 10020_2024_795_MOESM1_ESM.docx]

| Predicted genes |
| --- |
| COL1A1, COL1A2, COL4A2, COL12A1, COL3A1, COL5A2, COL14A1, COL6A3, COL6A2, BGN, THBS2, COL15A1, COL16A1, COL11A2, COL5A3, ELN, COL4A4, GDF5, COL8A2, LAMA4, COL7A1, COL4A6, COL24A1, DCN, FN1, LAMB1, COL21A1, MMP13, COL9A3, POSTN, MMP2, ACAN, LAMB2, COL19A1, LUM, FBLN5, COL23A1, COL27A1, IBSP, ADAMTS5, HSPG2, LAMC1, ADAMTS3, COL22A1, ADAMTS14, MMP3, COL6A6, LAMA5, TNC, LOXL1, FBLN2, TIMP3, FBLN1, ADAMTSL2, NID2, BMP7, FMOD, TGFB2, MFAP2, COL20A1, TGFB3, LOXL2, MMP1, SPP1, ADAMTS1, COL25A1, LAMA3, LTBP1, LAMC3, LAMB3, SPARC, ITGA10, ITGA11, COL28A1, COL13A1, SERPINH1, NID1, ADAMTS8, LAMA1, MFAP5, SOX9, MGP, OGN, EMILIN1, COMP, MMP9, CRTAP, ADAM12, THBS3, MMP14, BMP10, MFAP4, MMP12, THBS4, PCOLCE, MMP11, BMP1, LAMC2, MMP19, ADAMTS4, MMP15, ITGA1, ITGA2, INHBA, ADAMTS7, MMP8, THSD4, LAMB4, P3H1, COL17A1, FURIN, MMP10, ALPL, ADAMTS19, RUNX2, THBS1, FBN3, ADAMTS12, ADAMTS16, PLOD2, ADAMTS6, ADAMTSL5, ITGB4, HAPLN1, ADAMTS9, ITGA3, COLGALT2, COL26A1, P4HA1, TGFBI, TNXB, WNT5A, HTRA1, ADAMTSL3, TGFB1, LOX, ACTA2, LTBP3, ADAMTS13, SEMA5B, FRZB, ITGB6, ITGA7, B3GLCT, ADAMTS15, EFEMP2, SPON1, TIMP1, FSTL1 |
| The list contained top 5% (n=150) ranked genes from the inference algorithm model. |

**Supplementary Table T1** List of predicted gene by HumanNet V3

**Supplementary Table T2** List of the sequence of the guide RNA and the scrambled RNA used in the subtenon’s injection.

|  | Guide Sequence #1 | Guide Sequence #2 |
| --- | --- | --- |
| Scramble gRNA | GTGTAGTTCGACCATTCGTG | GTTCAGGATCACGTTACCGC |
| Thbs1 gRNA | CCAGTGCAAAGACGTCGATG | GTCATGCGTCCCGTCCGTGC |


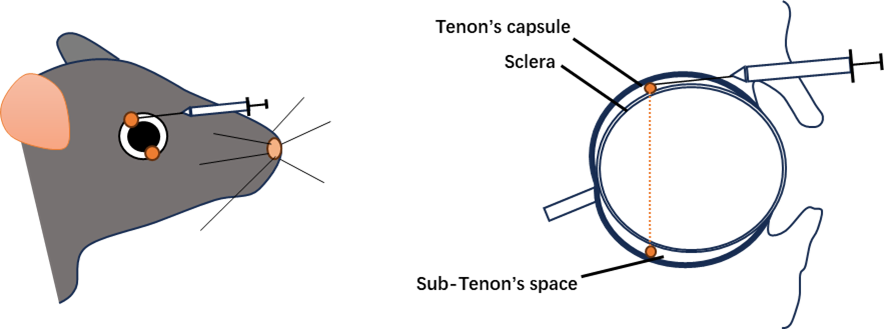


**Supplementary Figure S1** Subtenon's injections in mouse model (right eye as an example)

Subtenon's injections were administered at two locations around each eye, spaced 180 degrees apart avoiding blood vessels. The substance for injection, dissolved in sterile saline, is then slowly administered, and care is taken to avoid injury to the globe or surrounding tissues. The injections were a one-off for each group.


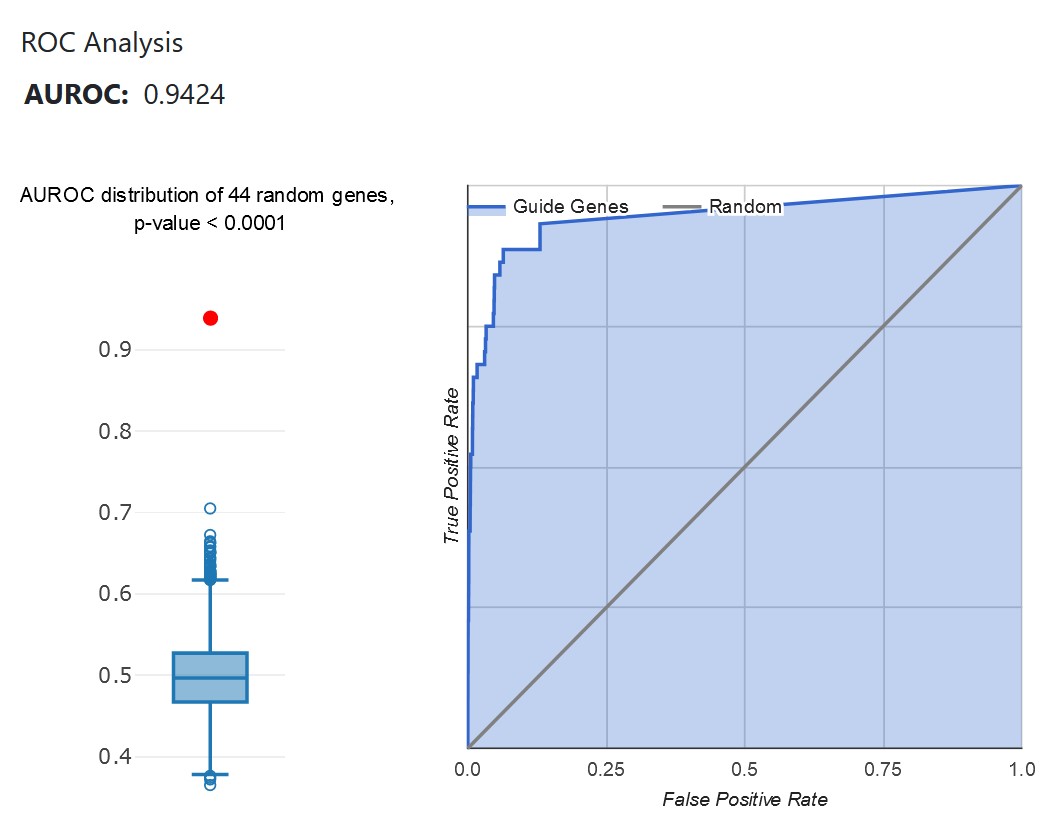


**Supplementary Figure S2** ROC analysis of network inference model.

The results of ROC analysis show that the AUROC score is 0.9424, indicating that the prediction tool has excellent analytical ability.


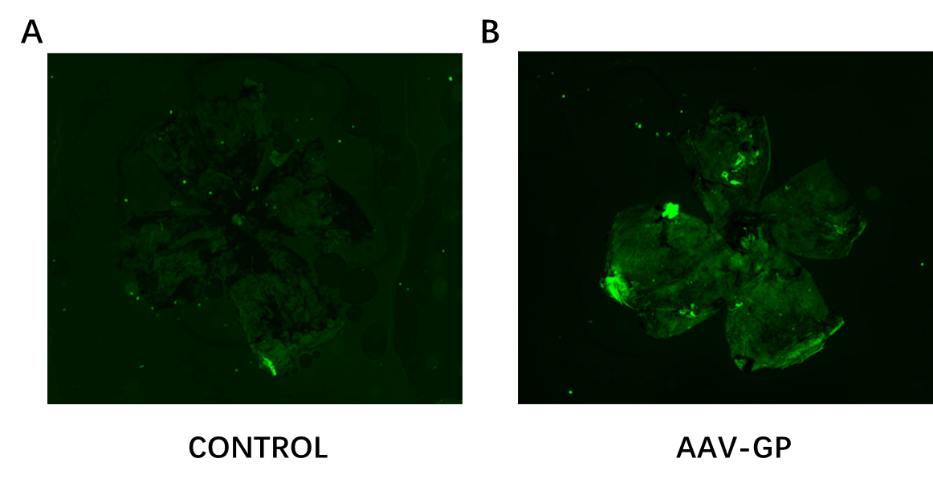


**Supplementary Figure S3** Distributions of green fluorescent protein (GFP) expression in the scleral whole-mount after AAV-GFP injection.

GFP expression (green) is distributed in the sclera of the AAV-GFP–injected eye (**B**) but not in control eye (**A**).
